# Mass media and risk factors for cancer: the under-representation of age

**DOI:** 10.1186/s12889-018-5341-9

**Published:** 2018-04-26

**Authors:** Sara Macdonald, Yvonne Cunningham, Chris Patterson, Katie Robb, Una Macleod, Thomas Anker, Shona Hilton

**Affiliations:** 1 Institute of Health & Wellbeing, General Practice and Primary Care, 1 Horselethill Rd, Glasgow, G12 9LX Scotland; 2 Institute of Health & Wellbeing, MRC/CSO Social and Public Health Sciences Unit, University of Glasgow, 200 Renfield Street, Glasgow, G2 3QB Scotland; 3 Institute of Health & WellbeingMental Health & Wellbeing, Gartnavel Royal Hospital, Administration Building, 1st floor, 1055 Great Western Road, Glasgow, G12 0XH Scotland; 4 Hull York Medical School, Allam Medical Building, Univrsity of Hull, Hull, HU6 7RX UK; 5 Adam Smith Business School, Gilbert Scott Building, University of Glasgow, Glasgow, G12 8QQ Scotland

**Keywords:** Media, Newspapwers, Risk factors, Ageing, Content analysis

## Abstract

**Background:**

Increasing age is a risk factor for developing cancer. Yet, older people commonly underestimate this risk, are less likely to be aware of the early symptoms, and are more likely to be diagnosed with advanced stage cancer. Mass media are a key influence on the public’s understanding health issues, including cancer risk. This study investigates how news media have represented age and other risk factors in the most common cancers over time.

**Methods:**

Eight hundred articles about the four most common cancers (breast, prostate, lung and colorectal) published within eight UK national newspapers in 2003, 2004, 2013 and 2014 were identified using the Nexis database. Relevant manifest content of articles was coded quantitatively and subjected to descriptive statistical analysis in SPSS to identify patterns across the data.

**Results:**

Risk was presented in half of the articles but this was rarely discussed in any depth and around a quarter of all articles introduced more than one risk factor, irrespective of cancer site. Age was mentioned as a risk factor in approximately 12% of all articles and this varied by cancer site. Age was most frequently reported in relation to prostate cancer and least often in articles about lung cancer. Articles featuring personal narratives more frequently focused on younger people and this was more pronounced in non-celebrity stories; only 15% of non-celebrity narratives were about people over 60. Other common risks discussed were family history and genetics, smoking, diet, alcohol, and environmental factors. Family history and genetics together featured as the most common risk factors. Risk factor reporting varied by site and family history was most commonly associated with breast cancer, diet with bowel cancer and smoking with lung cancer.

**Conclusion:**

Age and older adults were largely obscured in media representation of cancer and cancer experience. Indeed common risk factors in general were rarely discussed in any depth. Our findings will usefully inform the development of future cancer awareness campaigns and media guidelines. It is important that older adults appreciate their heightened risk, particularly in the context of help-seeking decisions.

## Background

The global burden of cancer is significant and incidence is increasing [1]. Much of the increase can be attributed to lengthening life expectancies, and increasing age is a risk factor for developing cancer [2, 3]. In the UK three quarters of all cancers are diagnosed in those over 60 years of age, and a third diagnosed in those over 75 [2]. The most common cancers in the UK are breast (55,222 new cases in 2014), lung (46,403 new cases in 2014), prostate (46,690 new cases in 2014), and colorectal (41,265 new cases in 2014), and the incidence of each is strongly correlated with increasing age [2]. When compared to analogous countries in Europe, Canada and Australia, older adults in the UK have markedly different survival outcomes [4] Poorer outcomes in the UK are at least in part attributable to later stage disgnoses [5].

Despite age emerging as an increasingly important risk factor for cancer, older adults commonly underestimate their risk, are less likely to be aware of early symptoms and are more likely to be diagnosed with cancer when it is in an advanced stage [6–8]. Robb [7] found that awareness of cancer risk factors and potential cancer symptoms decreased amongst those over 64, and more recently Quaife [9] found that the recognition of potential cancer signs such as persistent cough, unexplained bleeding or lump also decreased with age. While most national awareness campaigns in the UK, notably Be Clear on Cancer (BCC), are accessible to the entire population as a whole, they are specifically targeted at those over 50 [10]. Yet while early evaluations of awareness roadshows report an increase in overall awareness most are attended by those under 50 [11]. Emphasised that a third of breast cancer cases are diagnosed in women over 70, with the tag line ‘don’t assume you’re past it’ [12]. Early evaluation results suggest that awareness of cancer in older women increased across age groups and there has been a positive impact on urgent referrals from primary care [13].

Cancer awareness has been linked with helpseeking and presentation, and older adults are amongst those with lower awareness [9, 14]. In the UK it is estimated that many of the excess cancer deaths, when compared with the USA and Europe, occur in those over 75 and attribute the excess to late presentation in combination with less effective treatment [15]. Older adults feature prominently amongst emergency presentations for cancer [16, 17]. The proportion of patients presenting as an emergency rises with increasing age, reaching a peak in those aged over 85 years (40%) [18]. Around a fifth of cancer cases in England are diagnosed via emergency presentation and nearly a third of patients with colorectal cancer and over half of patients with lung cancer were admitted as emergencies [19], Moreover, it is known that patients admitted through this route have poorer outcomes [20].

Early diagnosis of cancer relies not only on prompt presentation of symptoms suggestive of cancer but also on national screening programmes. In the UK there are currently three national screening programmes for cancer – breast, bowel and cervical cancer – and all impose an upper age limit. While eligibility for automatic screening invitations stop at 70 and 74 for breast and bowel cancer respectively, those who wish to continue screening are encouraged to opt in to programmes [21]. Taken together, lack of awareness of risk and symptoms, later help-seeking, upper age limits within screening programmes and frequent diagnoses via emergency presentation suggest that the overall picture of cancer amongst older adults could be improved. Indeed, post-diagnostic treatment in older patients has also been highlighted as an area requiring additional research to improve overall outcomes [22].

Cancer risk factors include modifiable risks, such as smoking, alcohol, obesity and sun exposure as well as other risks such as increasing age and family history. Studies that seek to assess public awareness of such risk typically find high levels of awareness of the risks associated with smoking but lower awareness of other risk factors. Only 4% of those surveyed mentioned older age as a risk factor unprompted and this rose to 36% when prompted. International comparisons have shown that awareness of the links between cancer and increased age was lower in the UK, Canada and Australia as compared with Scandinavian countries [23]. Portrayal of risk in the media has tended to focus on lifestyle related factors [24] individual risks factors and/or individual cancer sites but equally has also demonstrated that risk messages in the media do not always reflect the range and importance of risk factors [25].

How people understand and act on information is often mediated and amplified through the mass media, therefore the media are a key influence on the public’s understandings and awareness of health issues, including cancer risk. Indeed the media are likely to be a key information source for those that do not seek ‘formal’ health information from other sources. The agenda-setting function of media is long-recognised and the media plays a role in shaping public understandings and health behaviours by choosing what news to report and how to report it [26]. As far back as the 1960s Cohen observed: “The press may not be successful much of the time in telling people what to think, but it is stunningly successful in telling its readers what to think about” [27]. While some media coverage will undoubtedly report formal awareness campaigns and be sanctioned by those with a specific public health remit, much of what appears in the media is ad hoc, reflects immediate events and stories. Such coverage can be powerful. The mass media contributes to individuals’ understandings of cancer incidence, risk, diagnosis, treatment and prognosis [28–33] and may provide ‘cues to action’ [34]. For example, media accounts of celebrities’ cancer experiences are well-recognised catalysts for public behaviours [35]: interest in cancer and early detection increased sharply following President Ronald Regan’s diagnosis of colon cancer in 1985 [36]; booking for mammography increased dramatically in the months following Kylie Minogue’s diagnosis of breast cancer in 2005 [37]; and the ‘Jade Goody Effect’ was shown to impact the uptake of cervical screening, particularly amongst younger women [38–41] and more recently the high profile preventive double mastectomy of Angelina Jolie, saw a considerable increase in BRCA testing in the United States immediately following an editorial in the New York Times [42]. Equally, however, the media has been criticised for omitting ‘mobilising’ information that, in theory, allows readers to act on existing attitudes [41]. Of specific interest here is the ‘Kylie Effect’ which led women to believe that breast cancer risk was highest in those under 70, and therefore suggest to older women that their age was associated with reduced risk [43].

Celebrity coverage notwithstanding, the media framing literature demonstrates that portrayals of cancer are often negative, promoting frames of dread and fear [24, 44], and frequently employ war and sporting metaphors [35, 45, 46]. Previous explorations of the representation of cancer in the media has compared the frequency of news coverage of specific cancer sites with their prevalence in the community and consistently demonstrates an overrepresentation of breast cancer and an underrepresentation of colorectal cancer [47].

Just as the media frames the representation of cancer the way in which older adults are portrayed in the media is not without critique. Many studies of a range of media - print media, television and advertising – typically conclude that older people are underrepresented [48, 49]. When older adults are portrayed two very differnet stereotypes dominate; one which focuses on a productive leisure filled retirement and the other more common frail and dependant older adults that require significant input from health and social care [48]. In general advertising has been found to be more positive and reflect the ‘golden ager’ [50], though Ylanne and collagues suggest that irrespective of the tone of the coverage ageing and health are inextricably linked [51]. Yet much of the coverage of ageing could be termed ‘apocalyptic demography’, which emphasises the burdensome nature of an ageing population [52, 53]. As previous research demonstrates the influence of the media on our broader understanding of health as well as shaping our cultural understanding of older age suggests there is merit in exploring the under-researched media representation of the association between older age and cancer. In particular we sought to explore the potential relationship between the portrayal of age in the news media and the help-seeking of older adults first by focusing on an analysis of the news media before carrying out a series of focus groups with older adults. Our aim in this paper is to explore the way in which the media represents the association between increasing age and cancer, among the four most common cancers: breast, colorectal, lung and prostate cancer. We opted to focus on those four cancer sites because a) we were mindful media representation varies by site and b) to provide a discrete focus for the study.We therefore explored the way in which cancer risk is represented in the UK print media, placing particular emphasis on ‘age’ as a risk factor. Here we present a manifest analysis of how cancer risk is represented and the place of age as a risk factor across the four most common cancers.

## Method

We chose eight UK daily national newspapers and their Sunday counterparts, representing three genres of newspapers: ‘serious’ – Daily Telegraph & Sunday Telegraph; ‘mid-market’ – Daily Mail & Mail on Sunday and the Daily Express & Sunday Express; and ‘tabloids’ – Daily Mirror & Sunday Mirror. These newspapers were selected as their readers tend to be older [54]. To provide longitudinal comparisons, we selected two time periods: a two-year period from 1st January 2003 to 31st December 2004, and a period ten years later from 1st January 2013 to 31st December 2014. Relevant newspaper articles were identified using the electronic database Nexis UK by searching for the terms *“cancer AND breast OR prostate OR lung OR colorectal OR bowel”* within article headlines. All articles were read (SH, SM, YC) to remove duplicate articles and exclude those that did not meet two inclusion criteria: the article must focus on breast, prostate, lung or colorectal cancer risk, and the article must be in news, feature or editorial format (therefore all letters from readers, obituaries, irrelevant articles and duplicate articles were excluded). We excluded articles, coded as ‘breakthrough’ stories that reported new or novel treatments but did not discuss risk. The search identified a total of 928 articles: 368 from 2003/4 and 560 from 2013/14. Following review, 128 were excluded due to either being duplicates of previously-accepted articles or failing to meet one or both of the inclusion criteria. This left 800 articles eligible for coding and analysis.

### Coding

To develop a coding frame, researchers (SH, SMcD, YC) read all the articles and identified categories and developed coding around our key a priori research questions: 1-How frequently is age represented as a risk factor for breast, prostate, lung, and colorectal cancers over time? 2-How does the frequency of age as a risk factor compare to other risk factors represented in breast, prostate, lung, and colorectal cancers?

The research team worked in close collaboration, checking and validating coding. Once all the data were coded, the data were entered into an SPSS file for descriptive statistical analysis to identify patterns across the data. To examine these patterns and provide more contextual explanations for differences across the data, free text was added into frameworks for textual analysis. Data were analysed by YC and CP. A binomial test was used to measure the significance of the difference in article count between the two time periods, and chi-square tests were used to test whether citations of specific risk factors differed significantly by time period.

Though we initially coded family history and genes separately we found that they rarely appeared individually and are likely to be conflated by readerships. Moreover, many of the articles presented risk factors, but few gave in-depth information on risks or associations. We therefore thought it necessary to distinguish between articles that ‘mentioned’ risk and those that ‘discussed’ risk or risk factors in more detail. Table 1 illustrates coding rationale for ‘mentioned’ and ‘discussed’.

**Table 1:** Examples of coding categorisation

Code	Newspaper	Headline	Text
Mentioned	Daily Mail	“Over the counter painkillers halve breast cancer risk”	“The anticancer effects of the drugs held true even when other factors that affect breast cancer risk, such as age, family history, weight and exercise, were taken into account” [55]
Discussed	Daily Telegraph	“Screening must not be stopped”	“Many women, however, are shocked to find that this automatic recall for scanning comes to an end just when the risk of developing breast cancer rises sharply. A number, including those quoted below, believe that what amounts to age discrimination must be stopped. The chance of developing breast cancer rises from one in 15,000 under the age of 25 to one in 50 by the age of 50. By 60, the risk is one in 23, rising to one in 10 by 80. Last year, the limit for automatic recall was raised from 65 to 70, and the aim is for all breast screening programmes to reach women in this age bracket by December.” [73]

## Results

As Table 2 shows of the 800 articles eligible for detailed coding and analysis, the majority were from mid-market-genre newspapers (275 from the Daily Mail and Mail on Sunday, 143 from the Express and Sunday Express), 202 from serious newspapers and 180 from tabloids. There was a marked difference in the number of articles focusing on each cancer site: 514 (64.3%) articles focused on breast cancer, 162 (20.3%) on prostate cancer, 63 (7.9%) on lung cancer, and 61 (7.6%) on colorectal cancer (Table 2). Breast cancer was the most common focus of articles in both 2003–4 (*n* = 226, 71.3%) and 2013–14 (*n* = 288, 59.6%). A total of 317 articles about cancer were published in 2003–4, and 483 in 2013–14 – a statistically significant (*p* < 0.001) difference of 52.4%. Risks were represented in just under half (49.5%) of all articles, and 25% of articles introduced more than one risk factor.

**Table 2:** Breakdown of stories over time by cancer site and newspaper genre

Time period	Cancer site	Publication genre						Total (*n* = 800)	
		Tabloid (*n* = 180)		Mid-market (*n* = 418)		Serious (*n* = 202)			
		*n*	%	*n*	%	*n*	%	*n*	%
2003–4(*n* = 317)	Breast	49	64.5	136	72.7	41	75.9	226	71.3
	Prostate	15	19.7	30	16.0	7	13.0	52	16.4
	Lung	6	7.9	11	5.9	3	5.6	20	6.3
	Colorectal	6	7.9	10	5.3	3	5.6	19	6.0
	Total	76	42.2	187	44.7	54	26.7	317	39.6
2013–14(*n* = 483)	Breast	70	67.3	135	58.4	83	56.1	288	59.6
	Prostate	14	13.5	58	25.1	38	25.7	110	22.8
	Lung	13	12.5	17	7.4	13	8.8	43	8.9
	Colorectal	7	6.7	21	9.1	14	9.5	42	8.7
	Total	104	57.8	231	55.3	148	73.3	483	60.4
All	Breast	119	66.1	271	64.8	124	61.4	514	64.3
	Prostate	29	16.1	88	21.1	45	22.3	162	20.3
	Lung	19	10.6	28	6.7	16	7.9	63	7.9
	Colorectal	13	7.2	31	7.4	17	8.4	61	7.6
	Total	180	100	418	100	202	100	800	100

### How frequently is age reported as a risk factor for breast, lung, prostate, and colorectal cancers over time? Fig. 1

Across all cancer sites age was briefly mentioned as a risk factor for 97 articles (12.1%), and discussed in 20 (2.5%) articles. In the articles about breast cancer (*n* = 514), age was mentioned in 59 (11.5%), and discussed in detail in 12 (2.3%) of them. Age was mentioned in 23 (14.2%) and discussed in 6 (3.7%) in the articles about prostate cancer. In the 61 articles about colorectal cancer, age was mentioned in 14 (23.0%) and discussed in one (1.59%). Finally, age was mentioned once (1.6%) and discussed once (1.6%) in the 63 articles about lung cancer. Comparing the two time periods, age was either mentioned or discussed as a risk factor in 59 (18.6%) articles in 2003–4 and 58 (12.0%) articles in 2013–14, a significant (chi-square with one degree of freedom = 6.68; *p* = 0.010) difference. However, this difference was not present in each cancer site; the proportions within articles about prostate, colorectal and lung cancer did not vary significantly (*p* = 0.762, *p* = 0.327, *p* = 0.833, respectively) between time periods, while the proportion of articles about breast cancer citing age (in either a brief mention or a more detailed discussion) varied significantly (chi-square with one degree of freedom = 10.84; *p* = 0.001) between 2003 and 4 (*n* = 44, 19.5%) and 2013–14 (*n* = 27, 9.4%).

**Fig. 1:**
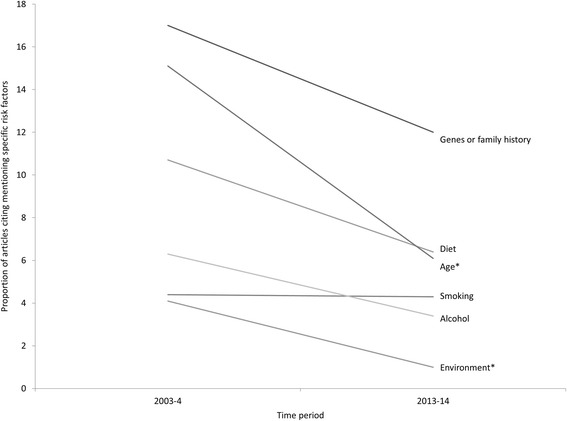
Changes in the reporting on risk factors between time periods

Stories that featured personal cancer narratives typically reported the age of individuals with cancer. We therefore considered how cancer more broadly was represented with regard to age. In the 121 articles describing celebrities with cancer, 43 (35.5%) were aged 61 years or older, 43 (35.5%) between 41 and 60 years and 35 (28.9%) 41 years or younger. In the 114 articles describing non-celebrities (which were largely about how these ‘ordinary’ people coped with cancer), 18 (15.8%) were aged 61 years or older, compared to 52 (43.9%) aged 41–60 and 46 (40.4%) aged 40 or younger.

### How frequently is age reported as a risk factor in comparison to other risk factors?

In addition to the 117 (14.6%) citations of age as a risk factor, articles reported on: diet (*n* = 122, 15.3%); family history and / or genes (*n* = 170, 21.3%); smoking (*n* = 63, 7.9%); alcohol (*n* = 62, 7.4%), and environmental factors (*n* = 27, 3.4%). Age was discussed as a risk factor in 20 (2.5%) articles. Diet (*n* = 37, 4.6%) and family history and/or genes (*n* = 58, 7.3%) were discussed more frequently, while alcohol (*n* = 15, 1.9%), and smoking (*n* = 15, 1.9%) were discussed less frequently. Table 3 outlines the frequency of brief mentions and more detailed discussions of different risk factors within articles focusing on one of the four cancer sites. Diet (including obesity) was particularly frequently cited as a risk factor for colorectal cancer, but never for lung cancer. Smoking was only mentioned in 63 stories, but, notably, was cited in 55.6% of articles about lung cancer. Similarly, environmental factors were mentioned only 27 times, and 13 of those mentions were associated with lung cancer. Age was mentioned in relation to each cancer site, but was only the most frequently cited risk factor in articles about prostate cancer (*n* = 29, 14.2%).

**Table 3:** Risk factors mentioned in articles about breast, prostate, colorectal and lung cancer

Risk factor	Cancer site															
	Breast (*n* = 514)				Prostate (*n* = 162)				Lung (n = 63)				Colorectal (*n* = 61)			
	Mentioned		Discussed		Mentioned		Discussed		Mentioned		Discussed		Mentioned		Discussed	
	*n*	%	*n*	%	*N*	%	*n*	%	*n*	%	*n*	%	*n*	%	*n*	%
Diet	57	11.1	29	5.6	11	6.8	7	4.3	0	0	0	0	17	27.9	1	1.6
Age	59	11.5	12	1.5	23	14.2	6	3.7	1	1.6	1	1.6	14	23	1	1.6
Genes and/or family history	88	17.1	41	8	15	9.3	10	6.2	4	6.3	2	3.2	5	8.2	5	8.2
Smoking	17	3.3	4	0.5	2	1.2	0	0	24	38.1	11	17.5	5	8.2	0	0
Alcohol	34	6.6	15	2.9	3	1.9	0	0	1	1.6	0	0	9	14.8	0	0
Environment	11	2.1	1	0.2	2	1.2	0	0	8	12.7	5	7.9	0	0	0	0

#### Breast cancer

Table 4 details how reporting on specific risk factors changed over time in each cancer site discussed. In addition to age (briefly mentioned 59 times, 11.5%; discussed in detail 12 times, 2.33%), articles frequently associated breast cancer with genes and/or family history (mentioned 88 times, 17.1%; discussed 41 times 8.0%), and diet (mentioned 57 times, 11.1%; discussed 29 times, 5.6%). While age (chi-square with one degree of freedom = 6.38; *p* = 0.012) and environment (chi-square with one degree of freedom = 6.54; *p* = 0.011) were mentioned more frequently in the first time period than the second within articles about breast cancer, genes and/or family history were mentioned more frequently in the second (chi-square with one degree of freedom = 4.55; *p* = 0.033). In addition to risk factors, mentions of risk reducers were coded; breast feeding was mentioned as a risk reducer for breast cancer 17 (3.3%) times and discussed in detail three (0.6%) times. Ninety-one articles (17.7%) mentioned breast cancer screening, and 25 (4.9%) of those mentioned some criticism of screening.

**Table 4:** Mention and Discussion of risk factors for cancer in newspaper articles

	2003–4 (*n* = 317)				2013–14 (*n* = 483)				Chi-square
	Mentioned		Discussed		Mentioned		Discussed		*p*-value
	*n*	1%	*n*	1%	*n*	2%	*n*	2%	
ALL SITES (*n* = 800)									
Age	48	15.1	11	1.4	49	6.1	9	1.1	0.034
Diet	34	10.7	17	2.1	51	6.4	20	2.5	0.94
Family history and/or genes	54	17	16	5	58	12	42	8.7	0.337
Smoking	14	4.4	8	1	34	4.3	7	0.9	0.127
Alcohol	20	6.3	9	1.1	27	3.4	6	0.8	0.672
Environment	13	4.1	2	0.3	8	1	4	0.5	0.034
BREAST (*n* = 514)									
Age	35	15.5	9	4	24	8.3	3	1	0.012
Diet	25	11.1	16	7.1	32	11.1	13	4.5	0.986
Family history and / or genes	44	19.5	14	6.2	44	15.3	27	9.4	0.075
Smoking	8	3.5	3	1.3	9	3.1	1	0.3	0.794
Alcohol	18	8	9	4	16	5.6	6	2.1	0.275
Environment	9	4	0	0	2	0.7	1	0.3	0.011
PROSTATE (*n* = 162)									
Age	8	15.4	2	3.8	15	13.6	4	3.6	0.766
Diet	6	11.5	1	1.9	5	4.5	6	5.5	0.099
Family history and / or genes	7	13.5	2	3.8	8	7.3	8	7.3	0.608
Smoking	0	0	0	0	2	1.8	0	0	0.328
Alcohol	1	1.9	0	0	2	1.8	0	0	0.963
Environment	2	3.8	0	0	0	0	0	0	0.038
LUNG (*n* = 63)									
Age	0	0	0	0	1	2.3	1	2.3	0.492
Diet	0	0	0	0	0	0	0	0	–
Family history and / or genes	1	5	0	0	3	7	2	4.7	0.492
Smoking	6	30	5	25	18	41.9	6	14	0.367
Alcohol	1	5	0	0	0	0	0	0	0.139
Environment	2	10	2	10	6	14	3	7	0.661
COLORECTAL (*n* = 61)									
Age	5	25	0	0	9	21.4	1	2.4	0.674
Diet	3	15	0	0	14	33.3	1	2.4	0.157
Family history and / or genes	2	10.5	0	0	3	7.1	5	11.9	0.656
Genes	0	0	0	0	2	4.8	3	7.1	0.333
Smoking	0	0	0	0	5	11.9	0	0	0.116
Alcohol	0	0	0	0	9	21.4	0	0	0.029
Environment	0	0	0	0	0	0	0	0	n/a

*P*-values are (*p* < 0.05)

#### Prostate cancer

Age (briefly mentioned 23 times, 14.2%; discussed in detail six times, 3.7%) was the risk factor most commonly linked with prostate cancer, while diet (mentioned 11 times, 6.8%; discussed seven times, 4.3%), genes and/or family history (mentioned 15 times, 9.3%; discussed ten times, 6.2%) also featured. The only risk factor that varied significantly between time periods within articles about prostate cancer was environmental factors (chi-square with one degree of freedom = 4.28; *p* = 0.038), which were mentioned just twice in 2003–4 and not at all in 2013–14. Forty-seven articles (29.0%) mentioned prostate cancer screening, of those 25 mentioned a criticism of screening.

#### Lung cancer

Smoking was the risk factor most frequently associated with lung cancer (mentioned 24 times, 38.1%; discussed 11, 17.5%), environment was mentioned eight times (12.7%) and discussed five times (7.9%), and genes and/or family history were mentioned four times (6.3%) and discussed twice (3.2%). Age was mentioned in one article (1.6%) and discussed in another (1.6%). No risk factors varied significantly by time period within articles about lung cancer. Only five articles (7.9%) mentioned screening for lung cancer, and one of those mentioned a criticism of screening.

#### Colorectal cancer

Diet was the most frequently mentioned risk factor (mentioned 17 times, 27.9%; discussed once, 1.6%), followed by age (mentioned 14 times, 23.0%; discussed once, 1.6%), family history and/or genes (mentioned five times, 8.2%; discussed five times, 8.2%) and alcohol (mentioned nine times, 14.8%; never discussed). Alcohol was the only risk factor that significantly (chi-square with one degree of freedom = 4.78; *p* = 0.029) varied by time period: no articles about colorectal cancer mentioned alcohol in 2003–4, while nine mentioned alcohol in 2013–4. Nineteen articles (31.1%) mentioned screening for colorectal cancer, and three of those articles mentioned a criticism of screening.

## Discussion

We conducted an analysis of media coverage of cancer risk associated with age over two distinct time periods, 2003–2004 and 2013–2014, and analysed four common cancers (breast, colorectal, lung and prostate). We found that more articles about cancer were published during the later period, though we did not find an equivalent increase in the number of articles linking cancer with increasing age. Fewer than 15% of articles documented an association between cancer and increased age, and only 2.5% of all articles discussed the importance of age in any depth. Between the two time periods the proportion of articles that mentioned age as a risk factor for prostate, lung and colorectal cancer remained relatively stable; but decreased significantly by 14% in articles about breast cancer. As well as age we also considered the range of other risk factors that appeared in the included articles. The most commonly cited risk factors varied by cancer site: breast cancer and genes and/or family history; prostate cancer and age; lung cancer and smoking; and colorectal cancer and diet. Among stories that featured personal narratives the main actors were typically under 60, and ‘non-celebrities’ were more likely to be younger still. We found that not only is age largely invisible but that risk more generally receives little attention. Yet, we found a discrepancy between the emphasis placed on some risk factors and the proportion of cancers attributed to such risks, for example the most common risk factor presented for breast cancer was family history and/or genes, which accounts for fewer than 5% of all breast cancers [55]. As a proportion of all stories, breast cancer featured more frequently than other sites, followed by prostate, lung and colorectal).

A major strength of our work is that while previous analyses of media representations of cancer have been conducted, few focus on how risk and specific risk factors are characterised. If we accept the role of the media as an information-giving vehicle it is useful to gain an insight into how the risk narrative is framed and we have demonstrated that certain cancer sites and risks are overrepresented at the expense of other relevant risk factors. It is also helpful to consider differences in two time periods ten years apart. However, we acknowledge that these two separate snap-shots cannot give evidence of continuous trends over time. Additionally, though we restricted our analysis to print media, which remain a popular and widespread source of information (especially among older adults), other media such as magazines, television broadcasts, radio, online news and social media are increasingly important. Moreover, we did not look at images or the size and prominence of headlines, but purely analysed the content of the article text. As is inherent to content analysis, we cannot determine how the public digest and act on the content analysed.

While analyses of the media representation of cancer are not new [56–58] previous studies have typically considered a more general depiction of the illness, and confirm the dominance of familiar metaphors such as those that use battle, combative and fighting language, and sporting analogies. Such a focus on intimate yet shared experiences of cancer confirms personalisation as a primary function of the media which permits the emphasis on the ‘human interest’ aspect of cancer stories [59]. Other content analyses have demonstrated that breast cancer, for example is consistently overrepresented in media coverage while other common cancers typically receive limited attention [28], resulting in a mismatch between incidence and news coverage [60]. Such analyses have also shown that media attention, in line with the common metaphors outlined above, invariably focuses on the treating and coping with cancer, rather than detecting and preventing it [28].

Our findings contribute to this body of evidence. We found breast cancer dominated media coverage and although such coverage is consistent with incidence, media attention was not commensurate with mortality. Lung cancer, which is responsible for the largest proportion of cancer deaths, was covered less frequently [28].

Previous studies have shown that media coverage has a demonstrable impact on behaviour. High-profile celebrity cases, such as Jade Goody’s diagnosis of cervical cancer and Kylie Minogue’s diagnosis of breast cancer were associated with increased screening uptake [35–42]. We propose therefore that a reasonable corollary to the mobilising effect of media coverage is that information that is missing or largely invisible is equally salient. As older adults—and associations between age and cancer—are routinely underrepresented older adults miss out on seeing the balance of information which could mobilise them to take up behaviour such as screening participation or timely symptom appraisal. Indeed older adults may feel reassured by their invisibility [61].

Age was largely invisible in stories containing personal narratives about individuals and as a risk factor in itself. Jones [62] concluded that most stories about breast cancer in Australian magazines did not feature women eligible for the breast-screening programme, and instead skewed coverage towards under-50s. Further, Chapman and colleagues’ [37] analysis of mammography requests following Kylie Minogue’s high profile breast cancer diagnosis found younger women requesting twice as many as eligible women.

We did however find that risk factors associated with lung, colorectal and prostate cancer (smoking, diet and age respectively) matched current awareness messages. However, the most common risk factor presented for breast cancer was family history and/or genes, which accounts for fewer than 5% of all breast cancers [63]. Moreover, given the dominance of breast cancer coverage, family history and genes assume a significant position in the overall presentation of risk. Our findings add weight to those of Walsh-Childers and colleagues [64] who considered both the substance and accuracy of the coverage of breast cancer in womens’ magazines and found that almost half of all stories included none of the ‘key facts’ about breast cancer. Associations between cancer and age featured in only 7% of included articles.

As well as inaccurate or missing risk information, it is worth emphasising that risk generally receives limited attention. We found few in-depth discussions of risk factors and instead they were merely ‘mentioned’, appearing in list-like form. Although the risk factors are accurate a surface approach fails to capture the complexity in the risk message. Our findings contribute to a growing body of work [28, 65] that considers the way in which the media presents cancer risk and deals with uncertainty. Short and brief snippets of information are likely to induce fatalistic beliefs and result in information overload thus leading to conclusions that ‘everything causes cancer’ [66]. Too much poor quality information leads to confusion about the appropriate advice to follow and the belief that little can be done to prevent cancer. Presenting scant information that aims to maximise the coverage of risk factors, irrespective of accuracy, may therefore be counter-productive. Conversely awareness interventions that are targeted at specific high risk groups produce positive results and in particular have a positive impact on early help-seeking [67–69]).

## Conclusion

*Although the risk of cancer increases as we get older, this doesn’t mean that you will definitely get cancer at some point. But it does mean that being aware of changes in your body, and going to the doctor if you notice anything unusual or that doesn’t go away, is even more important as we age.* [70]

As the above extract from Cancer Research UK’s website suggests older adults should be vigilant about cancer yet this is not reflected in the news media coverage of cancer risk. Taken together invisibility, inaccuracy and information overload build a skewed picture that cancer is a disease which affects younger people. Moreover, we demonstrate that family history of, or a genetic predisposition to cancer dominates aetiological explanations. Older adults with no family history may understandably underestimate their heightened risk. While focusing on age as a risk factor in the conventional sense, insofar as age is not modifiable, may be seen as problematic there are valid reasons for exploring the representation of the associations between ageing and cancer. Older adults experience poorer cancer outcomes, have lower awareness of risk and symptoms and are more likely to experience late stage diagnosis. Symptom appraisal studies have shown that older adults often attribute symptoms to natural consequences of ageing and therefore tend to seek help less promptly [71, 72]. Media coverage that fails to present a balance of risk may contribute to symptom misattribution. Encouraging older adults to consider cancer risk, and therefore appraise symptoms and bodily sensations in that context, may prompt earlier presentation and consequently impact on early detection.

Common cancer stories, although skewed towards younger age groups, tend to emphasise the random and unpredictable nature of the disease by reinforcing the ‘atypical case’. Accurate risk messages are therefore diluted and prevention strategies may feel unattainable. This may be particularly relevant for older adults who believe that there is little value in adopting or maintaining healthier behaviours. While it may be naïve and unreasonable to expect the media to shift away from its primary human interest function to become a mouthpiece for health educators, our findings have implications for those committed to awareness raising and information giving across the population. There is a pressing need to present a balanced and accurate representation of the sub-groups of the population most at risk of cancer. Older adults rarely see themselves represented among those that are affected by cancer, and therefore education and awareness raising messages need to counteract and re-balance messages. Stakeholders (including cancer research organisations and charities and representative groups for older people) should consider collaborating with media organisations and journalists to understand each other’s values. Indeed the co-production of reporting guidelines that balance the need for the media to focus on human interest stories while simultaneously representing a more convincing cancer picture is strongly indicted by our findings. This could only increase the quality of the journalism, and potentially enhance awareness of risk factors among readers.
